# Anemia in Early Childhood and Associated Factors in Ecuador: An Analysis From a Health Social Determinants Model

**DOI:** 10.1002/fsn3.70805

**Published:** 2025-08-19

**Authors:** María F. Rivadeneira, Nataly Cadena

**Affiliations:** ^1^ Facultad de Medicina, Instituto de Salud Pública Pontificia Universidad Católica del Ecuador Quito Ecuador; ^2^ Facultad de Medicina, Maestría en Epidemiología Para la Salud Pública Pontificia Universidad Católica del Ecuador Quito Ecuador

**Keywords:** anemia, cross‐sectional study, health inequalities, social determinants of health

## Abstract

Anemia is a problem highly prevalent in children associated with multiple factors. This study aims to identify factors associated with anemia in children under 5 years of age in Ecuador, based on the social determinants of health model. A secondary database study was conducted with information from 18,688 children aged 6–59 months included in the National Survey of Child Malnutrition 2022–2023. Anemia was defined by hemoglobin levels adjusted for age and height at sea level. Bivariate and multivariable Poisson regression for complex samples were used to calculate prevalence ratios with 95% confidence intervals. Anemia was found in 46.4% (95% CI 44.5–48.4) of children aged 6–23 months and 39.8% (95% CI 38.2–41.5) of those aged 24–59 months. For the 6–23‐month age group, the prevalence of anemia was higher in the lowest economic quintiles 1 and 2 (PR 1.26; 95% CI 1.05–1.51; PR 1.31; 95% CI 1.09–1.56), in those with latrines compared with toilets (PR 1.37; 95% CI 1.05–1.77), in children of adolescent or younger mothers (PR 1.29; 95% CI 1.08–1.53; PR 1.19; 95% CI 1.08–1.31), and in those with recent infectious diseases (PR 1.13; 95% CI 1.03–1.24). For the 24‐ to 59‐month‐old group, the prevalence of anemia was higher in males than in females (PR 1.10, 95% CI 1.03–1.18), in indigenous people and Afro‐Ecuadorians compared to mestizos or whites (PR 1.11, 95% CI 1.01–1.23; PR 1.35, 95% CI 1.19–1.53). Children of mothers with no education or only primary education had a 15% higher prevalence of anemia (PR 1.15, 95% CI 1.03–1.29) compared with children of mothers with a university education. In conclusion, gaps were found by gender, ethnicity, and socioeconomic status associated with the prevalence of anemia in children. A comprehensive approach is needed to reduce the prevalence of anemia in childhood.

## Graphical abstract

Four out of 10 children under 5 years of age in Ecuador suffer from anemia. Structural determinants of health, such as poverty, low maternal education, belonging to indigenous and Afro‐descendant ethnic groups, and intermediate determinants, such as access to food, infectious diseases, and sanitation and housing conditions, showed a significant association with anemia. This suggests the importance of policies aimed at improving living conditions, access to food, and the prevention of infectious diseases in children.

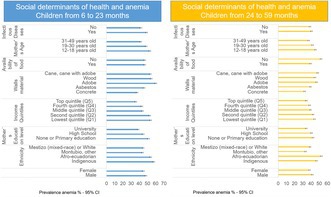

## 1 Introduction

Anemia is a relevant public health problem, with a high prevalence worldwide. In 2021, the global prevalence of anemia across all ages was 24.3%; however, for children under 5 years of age, the prevalence was 41.4%, almost double that of the general population (Gardner et al. 2023). It has been shown that children with hemoglobin levels below the optimal range are more likely to suffer from diseases and infections, as well as higher mortality (Scott et al. 2014). Anemia worsens the living conditions of the children because of lower cognitive and motor development (Larson et al. 2019). Likewise, it is estimated that iron deficiency anemia caused 52.0 million YLDs in 2021, accounting for 5.7% of all YLDs (Gardner et al. 2023).

Anemia is a multifactorial problem, with a complex interaction between nutrition, infectious diseases, and other factors, including socioeconomic and environmental factors (Balarajan et al. 2011). This complexity poses a major challenge in terms of establishing prevention and control measures by countries. In addition, it has been found that, over time, countries with greater socioeconomic inequalities fail to significantly reduce the prevalence of anemia, especially severe anemia (Yang et al. 2018).

The social determinants of health model provides a framework that could be useful for analyzing the complexity of factors associated with anemia. Social determinants of health could be defined as those “social conditions that affect health and that can potentially be altered by informed action” (Krieger 2001). The social determinants model recognizes the relationship between social structure and health/disease, considering biological (genetics, age, etc.), material, psychosocial, and behavioral elements (Marmot and Wilkinson 2005). This model includes structural and intermediate determinants of health. Structural determinants refer to the socioeconomic and political conditions that shape the social structure, while intermediate determinants are factors more closely related to the individual and their health experience (Marmot and Wilkinson 2005). Structural determinants include elements such as gender, socioeconomic status, and education; while intermediate determinants consider the home environment or material conditions, health behaviors, biological and psychosocial factors, and access to health services (Solar and Irwin 2010).

Ecuador is a country located in the Andes of South America, characterized by significant social inequality (Altmann 2024). According to data from the World Health Organization (WHO), in 2019, the prevalence of anemia in children aged 6–54 months reached 23.5% (95% CI 10.4%–39.7%), placing the country in a moderate prevalence of anemia (WHO 2021). This study aimed to identify the prevalence of anemia in children under 5 years of age and its associated factors, based on the model of social determinants of health.

## 2 Methods and Materials

### 2.1 Study and Sample

This cross‐sectional study utilizes a secondary database from the National Survey on Child Malnutrition (ENDI 2022–2023, first round), which aims to provide continuous, up‐to‐date information for the annual monitoring of Chronic Child Malnutrition (CCM) in Ecuador. ENDI is designed to produce nationally representative estimates, with an equitable distribution of samples across the country, conducted by the National Institute of Statistics and Census of Ecuador. The sampling is stratified two‐stage probability that includes: stratification of Primary Sampling Units (PSUs) and random selection of households within the selected PSUs. In the first stage, PSUs (usually geographic areas) are selected using probability sampling with probability proportional to size (PPS). This means that larger PSUs (with more households) are more likely to be selected. In the second stage, a fixed number of households are randomly selected within the selected PSUs. In the ENDI, eight households are selected per PSU. The sampling is conducted in two stages: (i) listing, which involves updating the sampling frame to ensure it includes the target population (children under 5 years old); and (ii) the collection of specific information from households within the selected Primary Sampling Units (PSUs) that contain the target population (Mendoza et al. 2023). ENDI 2022–2023 investigated 22,334 households nationwide, covering all 24 provinces of the country (Mendoza et al. 2023).

ENDI includes information from the children under 5 years of age, their mothers, and their households. For this study, data were included from children aged 6–59 months with available information on hemoglobin levels, and whose mothers had responded to the survey containing information on housing, socioeconomic, and health conditions.

Children who refused to have their hemoglobin tested and those with incomplete information about their mothers were excluded. For this study, data from 20,548 children aged 6–59 months included in the ENDI were reviewed. However, complete information and hemoglobin levels were only available for 18,688 children.

Personnel with expertise in fieldwork were trained to conduct surveys and blood hemoglobin testing. The collection of the hemoglobin sample was standardized and followed international guidelines (Mendoza et al. 2023). The equipment used for this process included: (a) Hemoglobinometer, a device utilized in other countries in the region for detecting anemia and employed in health establishments within the country; (b) Microcuvettes; and (c) Lancets, both pediatric and adult. Hemoglobin testing was conducted on children aged 6–59 months and women with children under 5 years old. Blood samples were collected with the consent of the caregiver or mother (Mendoza et al. 2023).

The methodology, datasets, and results of ENDI 2022–2023 can be accessed at https://www.ecuadorencifras.gob.ec/encuesta_nacional_desnutricion_infantil/ (Instituto Nacional de Estadísticas y Censos 2023).

### 2.2 Variables and Measures

The variables analyzed in this study were as follows.

#### 2.2.1 Dependent Variable: Anemia

The variable “Anemia” was constructed using the new WHO guidelines for diagnosing anemia in individuals and populations, published on March 8, 2024 (WHO 2024). According to these guidelines, the hemoglobin cutoff for defining anemia in children aged 6 to 23 months is now 10.5 g/dL, rather than the previous threshold of 11 g/dL. In addition, hemoglobin values now include adjustments beginning at 500 m rather than 1000 m at sea level, with reduced adjustments for populations above 3500 m and greater adjustments for elevations between 500 and 3000 m (WHO 2024; Vásquez‐Velásquez et al. 2024). Based on these adjustments, anemia is defined as Hb ≤ 10.5 g/dL for children aged 6–23 months, and Hb ≤ 11 g/dL for children aged 24–59 months. The anemia variable was categorized into “Yes” (anemia) and “No” (no anemia).

#### 2.2.2 Social Determinants of Health

**FIGURE 1:**
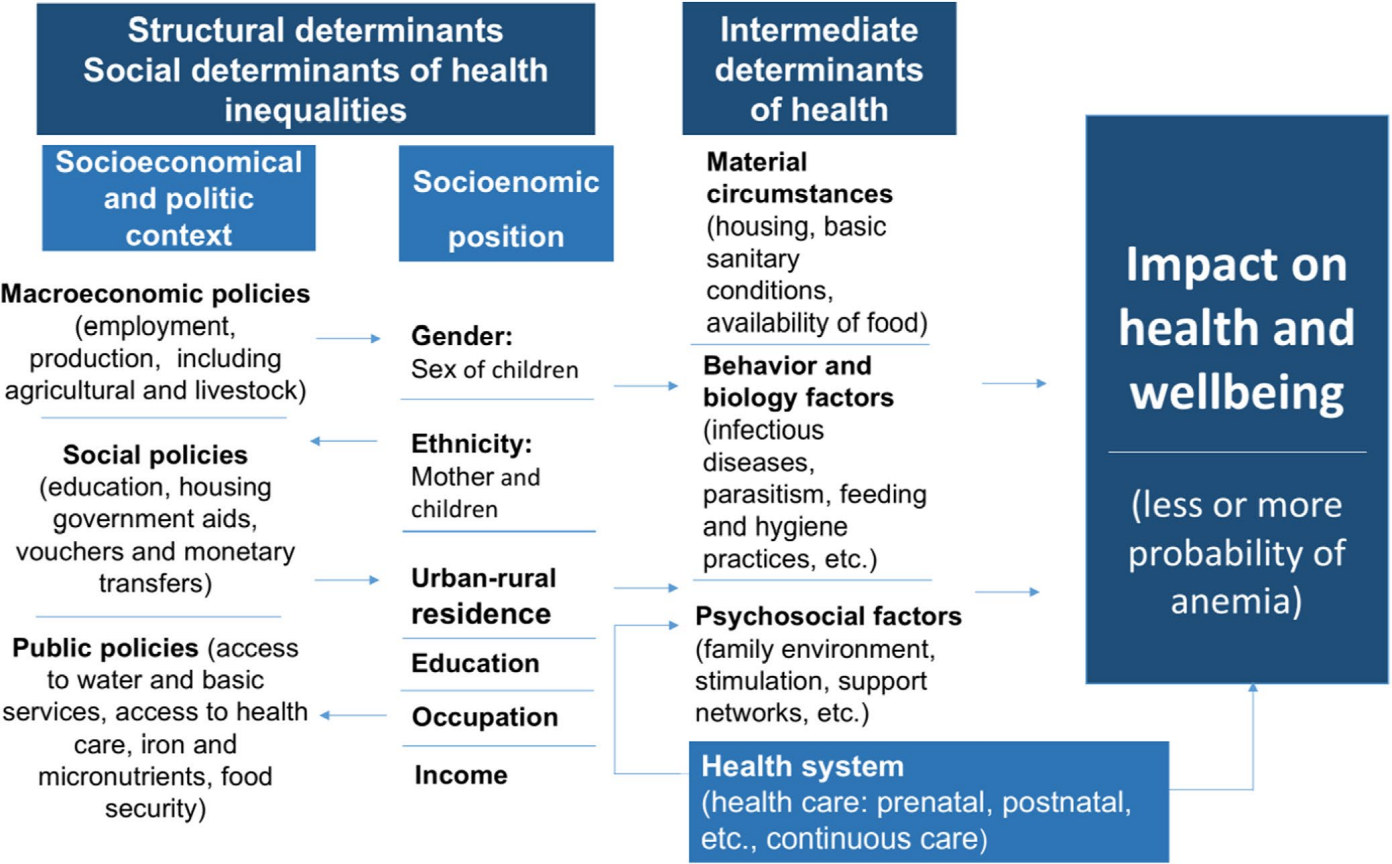
Social determinants of health model, applied to the analysis of anemia in children.

To measure social determinants of health, we identified variables representing social conditions that increase the likelihood of anemia, as suggested by prior studies (Ekholuenetale et al. 2022; Vart et al. 2015). Figure 1 integrates the components of the social determinants of health model applied to anemia. Following the model represented in Figure 1, we classify the ENDI variables as follows.

##### 2.2.2.1 Structural Determinants

- Gender: Child's biological sex (1 = male, 0 = female).
- Ethnicity: Self‐reported maternal ethnicity, recoded as Indigenous, Afro‐descendant, Montubio, Mestizo, White, or Other.
- Setting: Area of residence (1 = urban, 0 = rural).
- Education: Mother's education level, categorized as none/primary education, high school, and university.
- Income: Recoded into national income quintiles (Q1 = poorest, Q5 = richest).

##### 2.2.2.2 Intermediate Determinants

- Material circumstances:
  ○ Water *source:* Public network (tap water) vs. other (piped water, river water, spring water, irrigation ditch water, etc.).
  ○ Toilet facilities: Grouped as toilet and sewerage, toilet and septic tank, toilet and cesspool, latrine, or none.
  ○ Housing wall material: Concrete, asbestos, adobe, wood, cane/cane with adobe.
  ○ Availability of food: Based on binary questions: (1) concern about food shortage, and (2) whether the child went without food for an entire day (1 = yes, 0 = no).
- Behavioral and biological factors:
  ○ Mother's age: Categorized as 12–18, 19–30, and 31–49 years.
  ○ Infectious diseases: Presence of diarrhea or respiratory symptoms in the past 2 weeks (1 = yes, 0 = no).
  ○ Deworming: Whether the child had not received deworming medication in the past 6 months (1 = yes, 0 = no).
  ○ Feeding (for children aged 6–23 months only): Whether the child was breastfed during the first 6 months of life (1 = yes, 0 = no).
  ○ Hygiene practices: Handwashing with soap (1 = yes, 0 = no).
- Psychosocial factors:
  ○ Number of children under 5 in the Household: Grouped as 1–2, 3–4, and 5 or more.
  ○ Health system:
  ○ Place of delivery: Institutional (hospital or health center) vs. other.
  ○ Prenatal care: Fewer than 5 vs. 5 or more visits.
  ○ Iron consumption during pregnancy: Maternal receipt of iron supplements (1 = yes, 0 = no).
  ○ Iron supplementation for child: In the past 12 months (1 = yes, 0 = no).

### 2.3 Statistical Analysis

For the statistical analysis, the expansion factor was applied to the sample according to the methodology proposed in ENDI 2022–2023 (expanded sample) (Mendoza et al. 2023). Since factors related to anemia may exhibit different patterns in children under 2 years of age and those older (Cardoso et al. 2012), it was decided to stratify the sample and subsequent data analysis into: (a) children from 6 to 23 months, and (b) children from 24 to 59 months. Descriptive statistics were used to calculate the prevalence of anemia and 95% confidence intervals in both groups. Frequencies and percentages of anemia were calculated according to social determinants of health, following the theoretical model proposed for the analysis (Figure 1).

Bivariate and multivariable Poisson regression analyses for complex samples were conducted to assess the association between anemia and social determinants of health. The variables included in the bivariate analysis also followed the theoretical model shown in Figure 1. Considering the study design, prevalence ratios (PRs) were obtained with their respective 95% confidence intervals (95% CI). Tamhane et al. suggest the calculation of PR for population‐based cross‐sectional studies (Tamhane et al. 2016). Variables significantly associated with anemia, with a *p*‐value < 0.20, were retained for the multivariable analysis, as proposed by Victora et al. (1997). In the multivariable analysis, after adjusting for confounding factors, variables with a *p*‐value < 0.05 were considered statistically significant.

**FIGURE 2:**
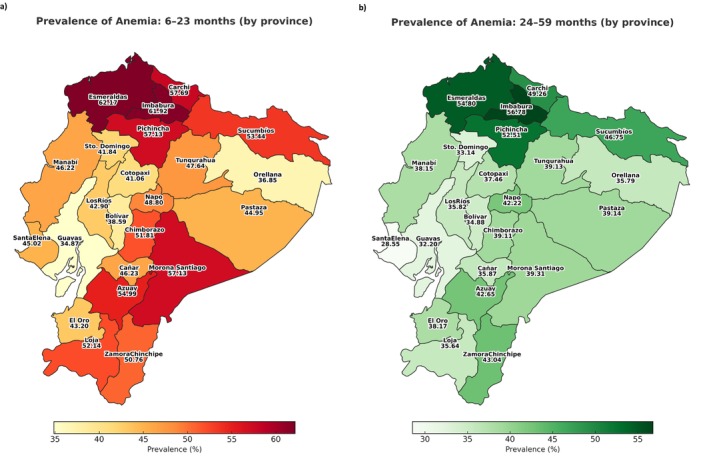
Prevalence of anemia (%) in: (a) Children from 6 up to 23 months of age, and (b) children from 24 up to 59 months of age by province. ENDI‐Ecuador, 2022. (Expanded sample *N* = 18,688).

Statistical analysis was conducted using STATA 18 software. In addition, two geographic maps were created using Tableau Public to illustrate the prevalence of anemia by age group and province (administrative unit in the country) (Figure 2).

## 3 Results

### 3.1 Characteristics of the Sample

Stratified results are presented for two age groups: 6–23 months and 24–59 months. Of the 18,688 children included in the study, 6140 children (32.9%) were aged 6–23 months, while 12,548 children (67.1%) were aged 24–59 months. The characteristics of the sample are detailed in Table 1.

**TABLE 1:** Characteristics of the sample of children aged 6 to 59 months. National survey on child malnutrition (ENDI), Ecuador, 2022 (expanded sample *N* = 18,688).

	6 up to 23 months of age		24 up to 59 months of age	
	Frequency	Percentage	Frequency	Percentage
**Structural determinants of health**				
*N*	6140	32.9	12,548	67.1
Gender				
Male	3128	50.9	6213	49.5
Female	3012	49.1	6335	50.5
Ethnicity				
Indigenous	614	10.0	1063	8.5
Afro‐ecuadorian	355	5.8	700	5.6
Montubio, other	341	5.6	923	7.4
Mestizo (mixed‐race) or White	4830	78.7	9862	78.6
Setting				
Urban	3877	63.1	8175	65.2
Rural	2263	36.9	4373	34.9
Mother's Education level				
None or Primary education	1884	30.7	3968	31.6
High School	3167	51.6	6052	48.2
University	1090	17.7	2528	20.2
Income Quintiles				
Lowest quintile	1259	20.5	2330	18.6
Second quintile	1410	23.0	2667	21.3
Middle quintile	1392	22.7	2774	22.1
Fourth quintile	1142	18.6	2648	21.1
Top quintile	937	15.3	2129	17.0
**Intermediate determinants of health**				
*Material circumstances*				
Water source				
Public network (tap water)	3975	64.7	8317	66.3
Other	2166	35.3	4231	33.7
Toilet facilities				
Toilet and sewerage	3411	55.6	7111	56.7
Toilet and septic tank	1862	30.3	3842	30.6
Toilet and cesspool	288	4.7	527	4.2
Latrine	103	1.7	178	1.4
None	476	7.8	890	7.1
Housing Walls material				
Concrete	495	8.1	1092	8.7
Asbestos	4595	74.8	9534	76.0
Adobe	90	1.5	218	1.7
Wood	517	8.4	849	6.8
Cane, cane with adobe	444	7.2	854	6.8
Availability of food				
Yes	5857	95.4	12,105	96.5
No	283	4.6	443	3.5
*Behavior and biology factors*				
Mother's age				
12–18 years old	452	7.4	145	1.2
19–30 years old	3739	60.9	7054	56.2
31–49 years old	1950	31.8	5349	42.6
Infectious Diseases				
Yes	4075	66.4	8012	63.9
No	2066	33.6	4536	36.2
Deworming				
No	5346	87.1	6243	49.8
Yes	794	12.9	6305	50.2
Feeding (only for 6–23 months)				
No	3971	67.2	—	—
Yes	1943	32.9	—	—
Hygiene practices				
No	1336	21.8	2482	19.8
Yes	4805	78.2	10,066	80.2
*Psychosocial factors*				
Number of children in household				
1–2 children	4894	79.7	9874	78.7
3–4 children	1116	18.2	2453	19.5
5 or more children	130	2.1	222	1.8
*Health system*				
Place of delivery				
Institutionalized birth	5898	96.1	11,915	95.0
Not institutionalized birth	243	4.0	633	5.1
Number of prenatal visits				
Fewer than 5	719	11.8	1367	11.1
5 or more	5362	88.2	11,007	89.0
Iron consumption (during pregnancy)				
No	397	6.5	765	6.2
Yes	5685	93.5	11,609	93.8
Iron supplementation				
No	3702	60.3	9402	74.9
Yes	2438	39.7	3146	25.1
Dependent variable				
Anemia				
No	3289	53.6	7554	60.2
Yes	2852	46.4	4994	39.8

For children aged 6–23 months, the prevalence of anemia was 46.4% (95% CI 44.5–48.4); for children aged 24–59 months, it was 39.8% (95% CI 38.2–41.5). Figure 2 shows the prevalence of anemia by province in children under 5 years of age. A higher prevalence of anemia was observed in the northern provinces of the country, the inter‐Andean region, and the Amazon.

### 3.2 Prevalence of Anemia

**FIGURE 3:**
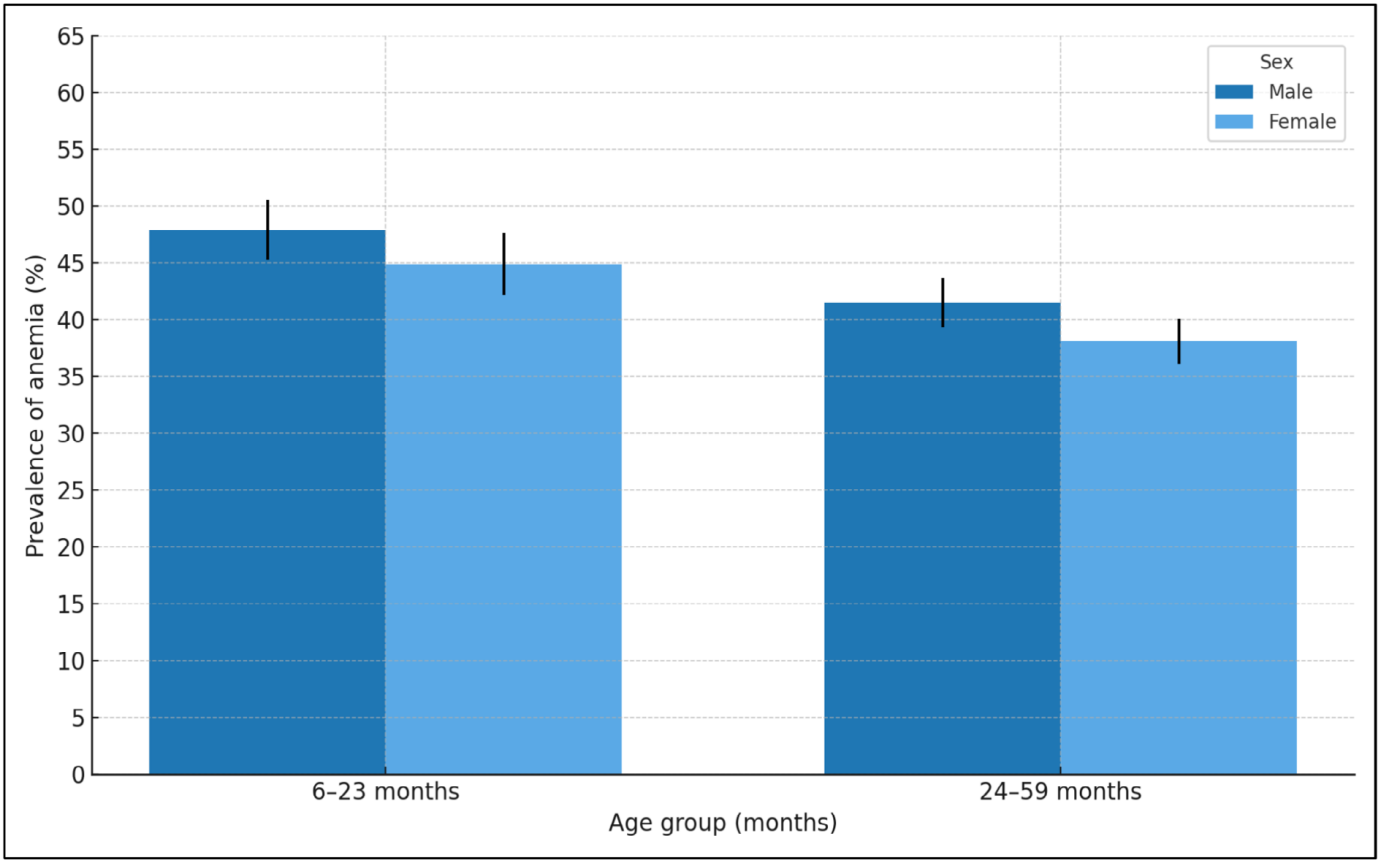
Prevalence of anemia (% and 95% CI) in children aged 6–23 months and 24–59 months, by sex. ENDI‐Ecuador, 2022–2023 (expanded sample, *n* = 18,688).

**FIGURE 4:**
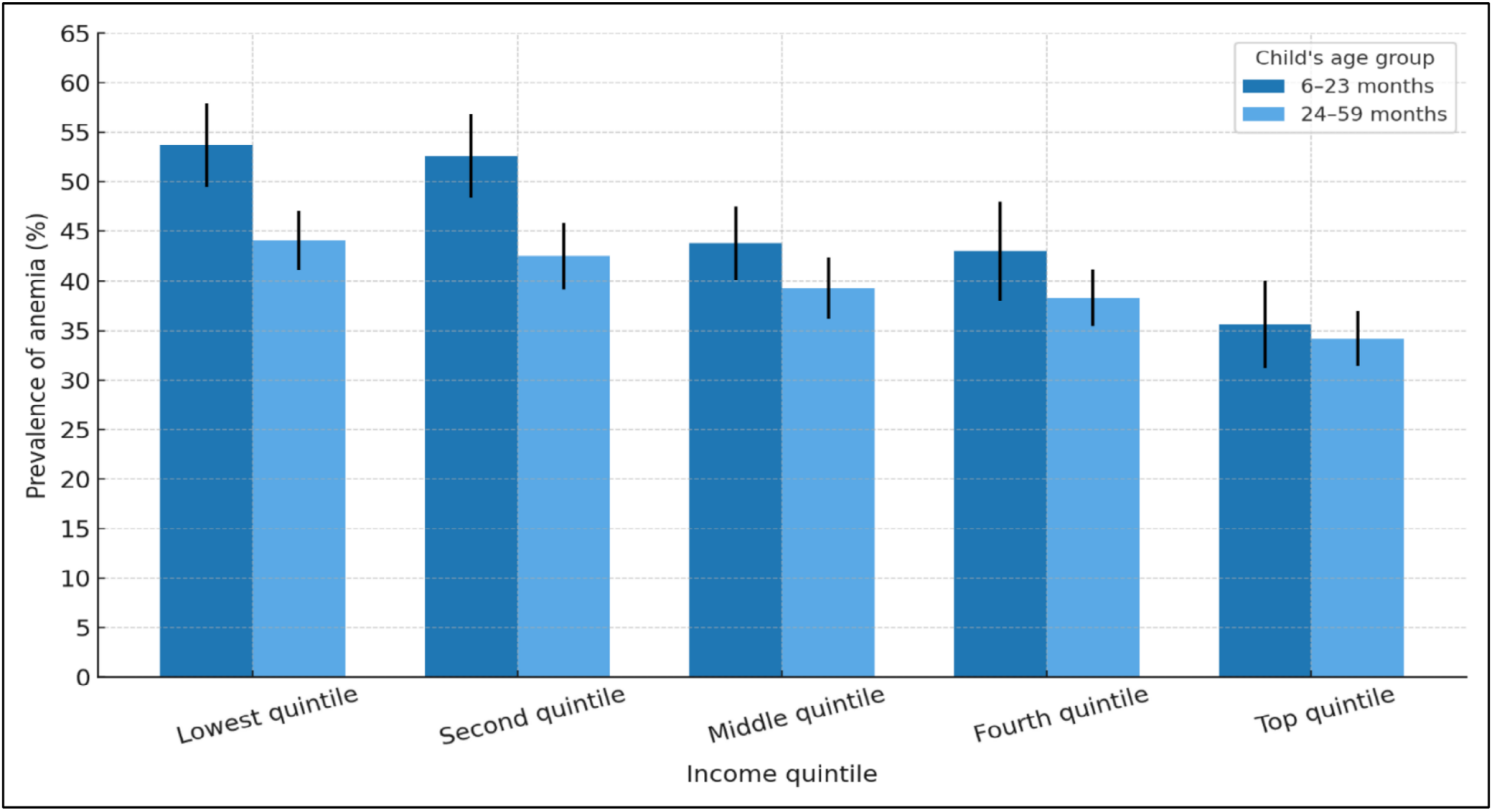
Prevalence of anemia by economic quintile (% and 95% CI) in children aged 6–23 months and 24–59 months. ENDI‐Ecuador, 2022–2023 (expanded sample, *n* = 18,688).

**FIGURE 5:**
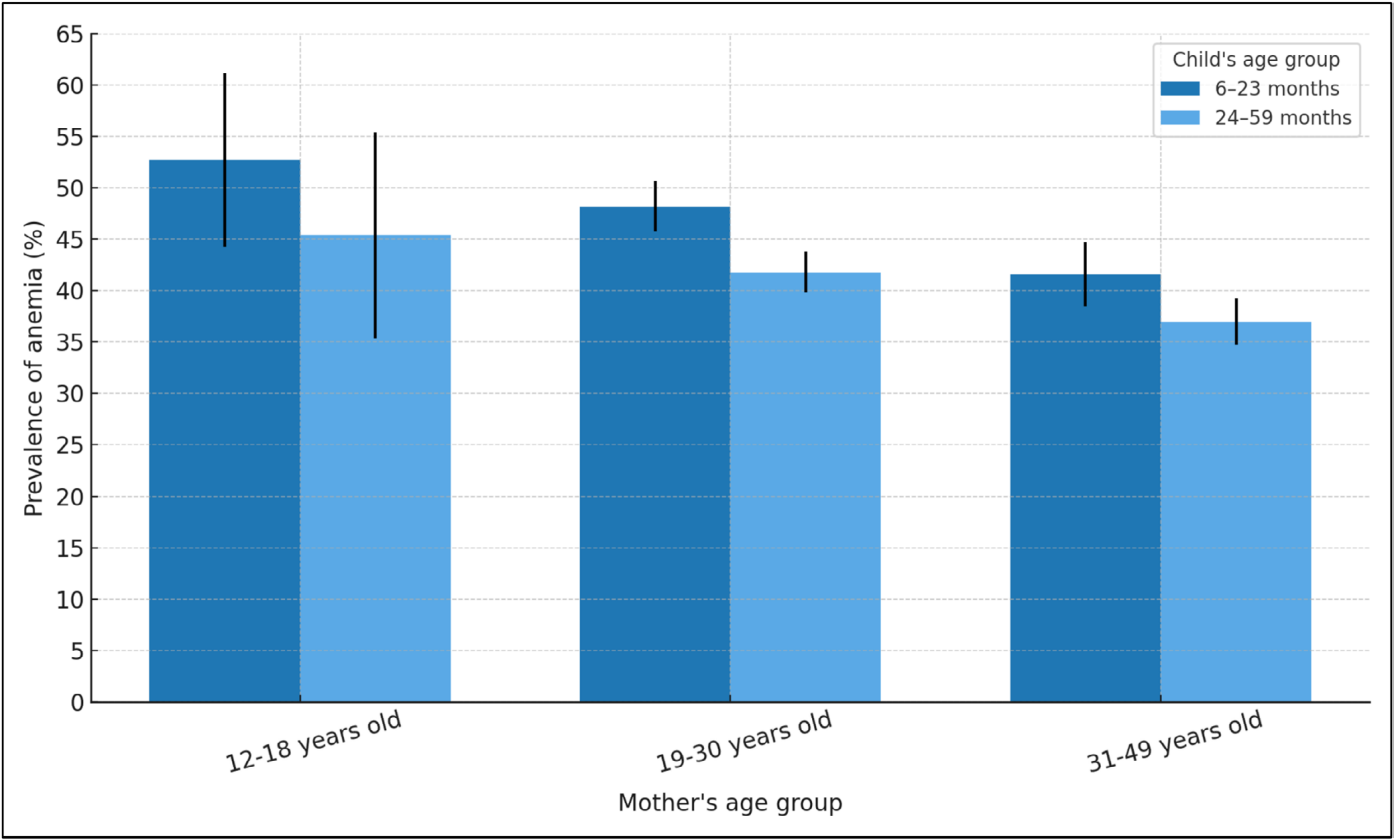
Prevalence of anemia (% and 95% CI) in children aged 6–23 months and 24–59 months, by maternal age group. ENDI‐Ecuador, 2022–2023 (expanded sample, *n* = 18,688).

Table 2 describes the prevalence of anemia with confidence intervals at 95%. For children aged 6–23 months, the percentage of anemia was 47.9% in males (95% CI 45.2–50.6) versus 44.9% (95% CI 42.1–47.6) in females. For children aged 24–59 months, the prevalence of anemia was 41.5% in males (95% CI 39.3–43.8) versus 38.1% in females (95% CI 36.1–40.1) (Figure 3). Regarding ethnic self‐identification, 57.6% (95% CI 53.00–62.00) of the indigenous children in the 6–23‐months group presented anemia. Anemia occurred in 52.6% (95% CI 46.4–58.6) of Afro‐Ecuadorian children in the 24–59 months group (Table 2). 49.7% (95% CI 46.6–52.8) and 40.4% (37.8–43.1) of children living in rural areas for each age group, respectively, had anemia. Anemia occurred in 51.1% (95% CI 50.5–57.7) and 44.1% (95% CI 41.1–47.1) of children of each age group, respectively, whose mothers have no education or only primary education. For both age groups, anemia was more prevalent among children from the lowest income quintile (53.7%, CI 49.5–57.7, and 44.1%, 95% CI 41.1–47.1, respectively) (Figure 4). Anemia occurred in 51.3% (95% CI 48.2–54.4) and 41.5% (95% CI 36.9–41.1) of children for each age group, respectively, whose households did not have public network water sources (Table 2). In children of adolescent mothers, anemia was present in 52.7% (95% CI 44.3–61.1) and 45.4% (95% CI 35.4–55.9) of each age group, respectively (Figure 5). Anemia was more common in children aged 6–23 months who had had infectious diseases in the previous 2 weeks (48.8%, 95% CI 46.5–51.1 vs. 41.8%, 95% CI 38.6–45.2 those who had not had infections). The prevalence of anemia was 48.8% (95% CI 46.2–51.3) in children aged 6–23 months who did not receive iron supplements (Table 2).

**TABLE 2:** Prevalence of anemia in children aged 6 to 59 months, according to social determinants of health. ENDI–Ecuador, 2022 (95% confidence interval for expanded sample *N* = 18,688).

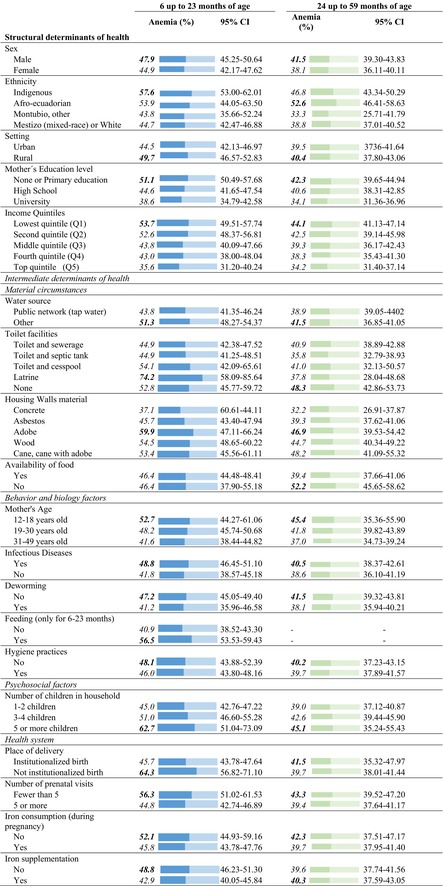

*Note:* Blue = prevalence of anemia for the categories indicated in Table 2, for children aged 6 to 23 months. Green = prevalence of anemia for the categories indicated in Table 2, for children aged 24 to 59 months.

### 3.3 Association Between Anemia and Social Health Determinants in Children Aged 6–23 Months

Table 3 shows the association between anemia in children and social health determinants from bivariate and multivariable analyses. For children aged 6–23months, no significant differences were found by sex (*p*‐value 0.06). Regarding ethnicity, indigenous children had a 1.29 times higher prevalence of anemia (95% CI 1.18–1.41) when compared to mestizos. However, statistical significance was not maintained in the multivariable analysis (*p*‐value 0.40). The rural area showed a 12% higher prevalence of anemia compared to the urban area (PR 1.12, 95% CI 1.03–1.21); although this did not remain statistically significant after adjustment in the multivariable analysis (*p*‐value 0.30). Children of mothers with no education or only basic education had a 40% higher prevalence of anemia than children of mothers with a university education level (PR 1.40, 95% CI 1.24–1.59); however, this was not statistically significant in the multivariable analysis (*p*‐value 0.24). Children from families in the lowest income quintiles had a higher prevalence of anemia (PR 1.51, 95% CI 1.30–1.74 and PR 1.48, 95% CI 1.27–1.72 for 1st and 2nd quintile, respectively), compared to those from the highest economic quintile. This result remained statistically significant in the multivariable analysis (*p*‐value < 0.05).

**TABLE 3:** Association between anemia and health social determinants. ENDI‐Ecuador, 2022 (Expanded Sample *N* = 18,688).

	6 up to 23 months of age				24 up to 59 months of age			
	Nonadjusted analysis	*p*	Adjusted analysis	*p*	Nonadjusted analysis	*p*	Adjusted analysis	*p*
	RP (95% CI)		RP (95% CI)		RP (95% CI)		RP (95% CI)	
**Structural determinants of health**								
Gender								
Male	1.07 (98.31–1.16)	0.119	1.08 (0.99–1.17)	0.063	1.09 (1.02–1.17)	0.011*	1.10 (1.03–1.18)	0.004**
Female	Reference		Reference		Reference		Reference	
Ethnicity								
Indigenous	1.29 (1.18–1.41)	0.000**	1.05 (0.94–1.17)	0.402	1.21 (1.11–1.32)	0.000**	1.11 (1.01–1.23)	0.027*
Afro‐ecuadorian	1.21 (0.99–1.47)	0.060	1.17 (0.99–1.38)	0.057	1.36 (1.20–1.53)	0.000**	1.35 (1.19–1.53)	0.000**
Montubio, other	0.98 (0.81–1.19)	0.841	0.94 (0.78–1.13)	0.491	0.86 (0.67–1.09)	0.216	0.84 (0.66–1.05)	0.125
Mestizo (mixed‐race) or White	Reference		Reference		Reference		Reference	
Setting								
Urban	Reference		Reference		Reference		Reference	
Rural	1.12 (1.03–1.21)	0.010*	0.93 (0.82–1.06)	0.305	1.02 (0.94–1.11)	0.592	0.97 (0.86–1.09)	0.609
Mother's Education level								
None or Primary education	1.40 (1.24–1.59)	0.000**	1.09 (0.94–1.27)	0.249	1.24 (1.12–1.37)	0.000**	1.15 (1.03–1.29)	0.016*
High School	1.15 (1.02–1.31)	0.022*	0.97 (0.84–1.11)	0.658	1.19 (1.08–1.31)	0.001**	1.12 (1.01–1.24)	0.040*
University	Reference		Reference		Reference		Reference	
Income Quintiles								
Lowest quintile (Q1)	1.51 (1.30–1.74)	0.000**	1.26 (1.05–1.51)	0.012*	1.29 (1.15–1.44)	0.000**	1.17 (1.03–1.34)	0.020*
Second quintile (Q2)	1.48 (1.27–1.72)	0.000**	1.31 (1.09–1.56)	0.003**	1.24 (1.10–1.40)	0.000**	1.16 (1.01–1.33)	0.030*
Middle quintile (Q3)	1.23 (1.05–1.45)	0.011*	1.14 (0.96–1.36)	0.136	1.15 (1.03–1.28)	0.015*	1.12 (0.99–1.26)	0.075
Fourth quintile (Q4)	1.21 (1.01–1.44)	0.040*	1.14 (0.94–1.37)	0.177	1.12 (1.00–1.25)	0.045*	1.08 (0.97–1.22)	0.172
Top quintile (Q5)	Reference		Reference		Reference		Reference	
**Intermediate determinants of health**								
*Material circumstances*								
Water source								
Public network (tap water)	Reference		Reference		Reference		Reference	
Other	1.17 (1.08–1.27)	0.000**	1.12 (0.98–1.28)	0.094	1.07 (0.99–1.15)	0.108	1.11 (0.99–1.26)	0.085
Toilet facilities								
Toilet and sewerage	Reference		Reference		Reference		Reference	
Toilet and septic tank	0.99 (0.90–1.10)	0.669	0.94 (0.83–1.07)	0.381	0.88 (0.80–0.97)	0.007**	0.83 (0.75–0.92)	0.001**
Toilet and cesspool	1.20 (0.96–1.51)	0.111	1.05 (0.82–1.33)	0.708	1.05 (0.80–1.27)	0.971	0.86 (0.68–1.08)	0.190
Latrine	1.65 (1.36–2.01)	0.000**	1.37 (1.05–1.77)	0.019*	0.93 (0.70–1.23)	0.589	0.73 (0.54–0.99)	0.054
None	1.18 (1.02–1.36)	0.028*	0.94 (0.78–1.13)	0.497	1.18 (1.04–1.34)	0.008**	0.98 (0.83–1.16)	0.824
Housing Walls material								
Concrete	Reference		Reference		Reference		Reference	
Asbestos	1.23 (1.02–1.49)	0.034*	1.16 (0.96–1.41)	0.121	1.22 (1.03–1.45)	0.021*	1.20 (1.01–1.41)	0.005**
Adobe	1.53 (1.19–1.97)	0.001**	1.24 (0.94–1.64)	0.135	1.46 (1.15–1.84)	0.002**	1.34 (1.04–1.73)	0.022*
Wood	1.47 (1.19–1.81)	0.000**	1.03 (0.76–1.39)	0.867	1.39 (1.14–1.69)	0.001**	1.30 (1.00–1.68)	0.047*
Cane, cane with adobe	1.44 (1.13–1.83)	0.003**	1.13 (0.85–1.50)	0.416	1.50 (1.21–1.86)	0.000*	1.51 (1.19–1.92)	0.001**
Availability of food								
Yes	Reference		Reference		Reference		Reference	
No	0.99 (0.82–1.21)	0.999	0.88 (0.73–1.06)	0.170	1.33 (1.16–1.51)	0.000*	1.21 (1.06–1.39)	0.005**
*Behavior and biology factors*								
Mother's Age								
12–18 years old	1.27 (1.05–1.53)	0.014*	1.29 (1.08–1.53)	0.005**	1.23 (0.97–1.55)	0.081	1.20 (0.95–1.51)	0.130
19–30 years old	1.16 (1.06–1.27)	0.001**	1.19 (1.08–1.31)	0.000*	1.13 (1.06–1.21)	0.000*	1.14 (1.05–1.22)	0.001**
31–49 years old	Reference		Reference		Reference		Reference	
Infectious diseases								
Yes	1.17 (1.06–1.28)	0.001**	1.13 (1.03–1.24)	0.009**	1.05 (0.96–1.14)	0.266	1.04 (0.96–1.12)	0.379
No	Reference		Reference		Reference		Reference	
Deworming								
No	1.15 (0.99–1.33)	0.062	1.18 (1.03–1.36)	0.016*	1.09 (1.02–1.17)	0.017*	1.07 (0.99–1.15)	0.052
Yes	Reference		Reference		Reference		Reference	
Hygiene practices								
No	1.05 (0.95–1.16)	0.381	0.97 (0.87–1.08)	0.587	1.01 (0.93–1.09)	0.791	0.93 (0.85–1.01)	0.091
Yes	Reference		Reference		Reference		Reference	
*Psychosocial factors*								
Number of children in household								
1–2 children	Reference		Reference		Reference		Reference	
3–4 children	1.13 (1.02–1.26)	0.01*	1.09 (0.97–1.22)	0.156	1.09 (1.00–1.19)	0.042*	1.06 (0.97–1.17)	0.189
5 or more children	1.39 (1.16–1.68)	0.000**	1.10 (0.87–1.40)	0.418	1.16 (0.92–1.46)	0.219	1.10 (0.85–1.41)	0.466
*Health system*								
Place of delivery								
Institutionalized birth	Reference		Reference		Reference		Reference	
Not institutionalized birth	1.41 (1.25–1.58)	0.000**	1.11 (0.96–1.29)	0.167	1.05 (0.89–1.22)	0.583	0.95 (0.82–1.09)	0.442
Number of prenatal visits								
Fewer than 5	1.26 (1.13–1.40)	0.000**	1.04 (0.93–1.17)	0.451	1.09 (0.99–1.21)	0.052	1.02 (0.93–1.11)	0.751
5 or more	Reference		Reference		Reference		Reference	
Iron consumption (during pregnancy)								
No	1.14 (0.98–1.32)	0.079	1.09 (0.94–1.25)	0.261	1.07 (0.94–1.20)	0.302	1.04 (0.93–1.18)	0.487
Yes	Reference		Reference		Reference		Reference	
Iron supplementation								
No	1.14 (1.04–1.24)	0.003**	1.08 (0.99–1.17)	0.095	0.98 (0.91–1.06)	0.681	0.94 (0.88–1.02)	0.142
Yes	Reference		Reference		Reference		Reference	

* Statistically significant difference, *p*‐value < 0.05.

** Statistically significant difference, *p‐*value < 0.01.

Regarding sanitation services, lack of access to the public drinking water network increased the prevalence of anemia by 17% (PR 1.17, 95% CI 1.08–1.27), not statistically significant in the multivariable analysis (*p*‐value 0.09). Children residing in households with latrine‐type sanitation facilities had a 1.65 times higher prevalence of anemia (95% CI 1.36–2.01) compared to those living in households with toilet facilities. This finding remained statistically significant in the multivariable analysis (*p*‐value 0.019). Those with adolescent mothers showed a higher prevalence of anemia (PR 1.27, 95% CI 1.05–1.53) compared to children of adult mothers. This result remained statistically significant in the multivariable analysis (*p*‐value 0.005). Children with infectious diseases in the past 2 weeks have a 1.17 times higher probability of anemia (95% CI 1.06–1.28) compared to those without these diseases; this result was statistically significant after adjustment for the other variables (*p*‐value 0.009). Children who have not been dewormed in the past 6 months have a 1.15 times higher prevalence of anemia (95% CI 0.99–1.33) compared to those who have been dewormed. This finding remained statistically significant after adjustment (*p*‐value 0.016) (Table 3).

As the number of children living in the household increased, a higher prevalence of anemia was observed (PR 1.13, 95% CI 1.01–1.26 for 3–4 children, and PR 95% 1.39, 95% CI 1.16–1.68 for 5 or more children). However, this finding was not statistically significant after adjustment (*p*‐value 0.156 and 0.418, respectively). Factors related to the health system, such as noninstitutionalized delivery, prenatal care visits under 5 years of age, and lack of iron supplementation in children, were associated with a higher prevalence of anemia (PR 1.41, 95% CI 1.25–1.58; PR 1.26, 95% CI 1.13–1.40; PR 1.14, 95% CI 1.04–1.24 for each of these factors, respectively). However, this association was not statistically significant in the multivariable analysis (*p*‐values 0.167, 0.451, and 0.095, respectively) (Table 3).

### 3.4 Association Between Anemia and Social Health Determinants in Children Aged 24–59 Months

For children aged 24–59 months, male children had a higher prevalence of anemia (PR 1.09, 95% CI 1.02–1.17) compared to female children. This difference was also statistically significant in the multivariable analysis (*p*‐value 0.004) (Table 3). Children of mothers belonging to ethnic minorities, such as indigenous and Afro‐Ecuadorian, had higher probabilities of anemia (PR 1.21, 95% CI 1.11–1.32 and PR 1.36, 95% CI 1.20–1.53, respectively), compared to mestizo (*p*‐value 0.027 and 0.00 in the multivariable analysis). Children whose mothers had no education or only secondary/high school education showed an increased prevalence of anemia (PR 1.24, 95% CI 1.12–1.37 and PR 1.19, 95% CI 1.08–1.31, respectively), compared to mothers with university education. This result was statistically significant in the multivariable analysis (*p*‐value 0.016 and 0.040, respectively). Lower income quintiles had a higher prevalence of anemia (PR 1.29, 95% CI 1.15–1.44 and PR 1.24, 95% CI 1.10–1.40), compared to the highest income quintile. This finding remained significant in the multivariable analysis (*p*‐value 0.02 and 0.03).

Regarding intermediate determinants of health, housing wall materials like asbestos, adobe, wood, and cane were associated with an increased probability of anemia (PR 1.22, 95% CI 1.03–1.45; PR 1.46, 95% CI 1.15–1.84; PR 1.39, 95% CI 1.14–1.69, and PR 1.50, 95% CI 1.21–1.86, respectively), compared to concrete walls. This finding remained significant in the multivariable analysis (*p*‐value 0.005, 0.022, 0.047, and 0.001).

Lack of availability of food increased the prevalence of anemia by 33% (PR 1.33, 95% CI 1.16–1.51) compared to those with access to food. Children of young mothers aged 19–30 years have a 1.13 times greater prevalence of anemia (95% CI 1.06–1.21) compared to children of adult mothers. These associations remained significant after adjustment (*p*‐value 0.001) (Table 3).

Factors related to the health system, such as noninstitutionalized delivery, prenatal care visits under 5 years of age, and lack of iron supplementation in children, were not associated with a higher prevalence of anemia in children aged 24–59 months.

## 4 Discussion

This study aimed to analyze the prevalence of anemia and associated factors in Ecuadorian children aged 6–59 months, based on a model of social determinants of health. The analysis was stratified into two age groups, 6–23 months and 24–59 months. Anemia was found in 46.4% of children aged 6–23 months (95% CI 44.5–48.4); 39.8% of children aged 24–59 months (95% CI 38.2–41.5). This data is similar to that reported in other studies conducted globally in children under 5 years of age (Gardner et al. 2023; Allali et al. 2017).

### 4.1 Structural Determinants of Health and Anemia

Structural determinants of health, such as lower economic income (Q1) showed an increase in the prevalence of anemia of 26% and 17% for children from 0 to 23 months and from 24 to 59 months of age, respectively (PR 1.26, 95% CI 1.05–1.51 and PR 1.17, CI 1.03–1.34, respectively, in the multivariable analysis, *p*‐value 0.020 and 0.030). Various studies have found that children from families with low socioeconomic status have a higher probability of anemia (Andriastuti et al. 2020; Yang et al. 2018; Sharma et al. 2020). Yang et al. (2018) found in their multinational study a 12% higher rate of anemia in children from the lowest socioeconomic stratum when compared to the highest. Lower economic access in families would be related to exposure to greater risk factors for anemia, such as low iron consumption, greater probability of infectious diseases, and less access to education and health (Yang et al. 2018; Sharma et al. 2020).

In this study, low maternal education, indigenous, and afro‐descendant children were associated with a higher prevalence of anemia among children aged 24–59months. Children of mothers with no education or only primary education had a 15% higher prevalence of anemia than children of university‐educated mothers (PR 1.15, 95% CI 1.03–1.29, *p*‐value 0.016, in the multivariable analysis). Maternal education has been a predominant factor in previous studies (Andriastuti et al. 2020; Yang et al. 2018; Sharma et al. 2020). A mother's educational level is closely related to the family's socioeconomic level, as well as to the mother's agency in the care, feeding, and health of young children (Chen and Li 2009). Higher education is associated with a better understanding of nutrition and health practices, which may contribute to the prevention of anemia, seek adequate medical care, and provide more nutritious food to their children (Rezaeizadeh et al. 2024). On the other hand, Indigenous and Afro‐Ecuadorian children had an 11% and 35% higher prevalence of anemia, respectively, than mestizos (PR 1.11, 95% CI 1.01–1.23 and PR 1.35, 95% CI 1.19–1.53, *p*‐value 0.027 and 0.000, in the adjusted analysis). The higher prevalence of anemia found in indigenous ethnicity and afro‐descendant children is consistent with previous studies (Rosas‐Jiménez et al. 2022; Leite et al. 2013; da Silva Ferreira et al. 2021). Indigenous and Afro‐descendent populations have higher risk factors for anemia due to several factors, including poor living conditions, limited access to health services, racism, and discrimination (Rosas‐Jiménez et al. 2022; da Silva Ferreira et al. 2021).

### 4.2 Intermediate Determinants of Health and Anemia

This study found a significant association between anemia in children and a lack of access to sanitation services, and worse housing conditions. Thus, the prevalence of anemia was 37% higher in children aged 0–23 months who had latrines compared to those who had toilets (PR 1.37, 95% CI 1.05–1.77, *p*‐value 0.019 after adjustment). The lack of sanitation services has been associated with a higher probability of infections and diarrhea, which would be related to a higher probability of anemia (Kothari et al. 2019). In children aged 24–59 months who lived in houses with asbestos walls, the prevalence of anemia was 34% higher compared to those who lived in concrete houses (PR 1.20, 95% CI 1.01–1.41, *p*‐value 0.005, after adjustment). Wooden walls were also associated with a higher prevalence of anemia in children aged 24–59 months (PR 1.30, 95% CI 1.00–1.68, *p*‐value 0.022, after adjustment). A previous study linked wooden walls with a higher prevalence of malaria (Mhelembe et al. 2025), which in turn has been associated with microscopic anemia (Chaves et al. 2025). On the other hand, the lack of availability of food increased the prevalence of anemia in children aged 24–59 months (PR 1.21, 95% CI 1.06–1.39, *p*‐value 0.005). In this regard, a higher probability of anemia has been found in children who cannot access a diverse diet (Majumder et al. 2025) and children from vulnerable environments, who have a high consumption of ultra‐processed foods (Queiroz et al. 2025). This result highlights the need for food safety protection policies and control of the consumption of ultra‐processed foods.

Regarding behaviors and biological factors, adolescent and younger mothers had more children with anemia. Among adolescent mothers (12–18 years old), children aged 6–23 months had a 29% higher prevalence of anemia compared to those born to mothers older than 30 years (PR 1.29 95% CI 1.08–1.53, *p*‐value 0.005). Among children aged 24–59 months, the prevalence of anemia was higher among children of young adult mothers (19–30 years old) (PR 1.14, 95% CI 1.05–1.22, *p*‐value 0.001). This finding has been well documented previously (Obasohan et al. 2022; Rammohan et al. 2025). Teenage mothers tend to have a higher prevalence of anemia, which is associated with a higher probability of anemia in their children (Rammohan et al. 2025). Furthermore, the mother's age is related to her level of knowledge and autonomy in making decisions about the care of her children.

On the other hand, in children aged 6–23 months, there was a higher prevalence of anemia in those who had had infections (PR 1.13, 95% CI 1.03–1.24, *p*‐value 0.009). These results coincide with previous studies (Pasricha et al. 2018; Ngure et al. 2014). According to them, infections would promote an inadequate enteric environment, with malabsorption of micronutrients and a greater state of inflammation, which would be related to a greater probability of anemia.

Factors related to the health services system, such as prenatal care and iron supplementation, did not show a significant association with anemia in the multivariable analysis. These findings highlight the importance of policies aimed at the comprehensive health of mothers and children, focused on reducing socioeconomic inequalities and improving living conditions and the sanitary and nutritional environment in which children grow and develop.

This study has several limitations. Being a cross‐sectional study, it does not allow establishing causal relationships. Some variables such as access to food, number of infections, and deworming could be subject to information biases by the mother. Moreover, the database does not allow evaluation of variables such as breastfeeding or consumption of iron‐rich foods, since the number of respondents was lower and did not allow generating a stable model. However, the sample is representative at the population level, and the analysis used in this study includes a broad approach to factors associated with anemia.

## 5 Conclusion

Four out of 10 children under 5 years of age in Ecuador suffer from anemia. This study found that structural determinants of health, such as poverty, low maternal education, and belonging to indigenous and Afro‐descendant ethnic groups, were significantly associated with the prevalence of anemia in Ecuador. Regarding intermediate determinants, a lack of access to food, poor sanitation and housing conditions, and infections were also significantly associated with anemia. Differences were found by age group: in the 6‐ to 23‐month age group, poverty (poorest quintiles 1 and 2), inadequate environment (use of latrines instead of sewage disposal), maternal age (adolescents and young adults), and infections in the last few weeks remained significantly associated with anemia after adjustment. Meanwhile, in the 24‐ to 59‐month age group, anemia was significantly associated with male sex, Indigenous and Afro‐Ecuadorian ethnicity, lower maternal education, poverty (poorest quintiles 1 and 2), and a lack of access to food. These findings emphasize the importance of a comprehensive approach, focused on the determinants of health, for the implementation of policies aimed at reducing anemia in children.

## Author Contributions

M.F.R. and N.C. designed the study. N.C. performed the data analysis. M.F.R. made the preliminary draft. N.C. made important contributions to the interpretation and discussion of the results. All authors made contributions to the draft. All authors agree with the information presented in this abstract.

## Ethics Statement

This research was approved by the Ethics Committee for Research in Human Beings of the Pontifical Catholic University of Ecuador, code 2018‐12‐MB.

## Conflicts of Interest

The authors declare no conflicts of interest.

## Data Availability

The data that supports the findings of this study are openly available in Instituto Nacional de Estadística y Censos. Encuesta Nacional sobre Desnutrición Infantil. Retrieved from: https://www.ecuadorencifras.gob.ec/encuesta_nacional_desnutricion_infantil/.
